# Immune niches in brain metastases contain TCF1+ stem-like T cells, are associated with disease control and are modulated by preoperative SRS

**DOI:** 10.21203/rs.3.rs-2722744/v1

**Published:** 2023-03-23

**Authors:** Caroline S. Jansen, Roshan S. Prabhu, Meghana S. Pagadala, Prasanthi Chappa, Subir Goyal, Chengjing Zhou, Stewart G. Neill, Nataliya Prokhnevska, Maria Cardenas, Kimberly B. Hoang, Jim Zhong, Mylin Torres, Suzanna Logan, Jeffrey J. Olson, Edjah K. Nduom, Luke del Balzo, Kirtesh Patel, Stuart H. Burri, Anthony L. Asher, Scott Wilkinson, Ross Lake, Kristin A. Higgins, Pretesh Patel, Vishal Dhere, Adam G. Sowalsky, Mohammad K. Khan, Haydn Kissick, Zachary S. Buchwald

**Affiliations:** 1 Department of Urology and Winship Cancer Institute, Emory University, Atlanta, GA, USA; 2 Southeast Radiation Oncology Group, Levine Cancer Institute, Atrium Health, Charlotte, NC, USA; 3 Biomedical Science Program, University of California San Diego, La Jolla, CA, USA; 4 Department of Radiation Oncology and Winship Cancer Institute, Emory University, Atlanta, GA, USA; 5 Department of Biostatistics and Winship Cancer Institute, Emory University, Atlanta, GA, USA; 6 Department of Pathology and Winship Cancer Institute, Emory University, Atlanta, GA, USA; 7 Department of Neurosurgery and Winship Cancer Institute, Emory University, Atlanta, GA, USA; 8 Department of Pathology, Nationwide Children’s Hospital, Columbus, OH, USA; 9 Kaiser Permanente, Atlanta, GA, USA; 10 Neuroscience Institute, Atrium Health, Charlotte, NC, USA; 11 Laboratory of Genitourinary Cancer Pathogenesis, National Cancer Institute, Bethesda, MD, USA; 12 Emory Vaccine Center, Department of Microbiology and Immunology, Emory University, Atlanta, GA, USA

## Abstract

The CD8^+^ T-cell response is prognostic for survival outcomes in several tumor types. However, whether this extends to tumors in the brain, an organ with barriers to T cell entry, remains unclear. Here, we analyzed immune infiltration in 67 brain metastasis (BrM) and found high frequencies of PD1^+^ TCF1^+^ stem-like CD8^+^ T-cells and TCF1^−^ effector-like cells. Importantly, the stem-like cells aggregate with antigen presenting cells in immune niches, and niches were prognostic for local disease control. Standard of care for BrM is resection followed by stereotactic radiosurgery (SRS), so to determine SRS’s impact on the BrM immune response, we examined 76 BrM treated with pre-operative SRS (pSRS). pSRS acutely reduced CD8^+^ T cells at 3 days. However, CD8^+^ T cells rebounded by day 6, driven by increased frequency of effector-like cells. This suggests that the immune response in BrM can be regenerated rapidly, likely by the local TCF1^+^ stem-like population.

## Introduction:

CD8^+^ T cell infiltration in primary and metastatic sites of several cancer types is associated with longer progression free survival, overall survival, and superior responses to immunotherapy.^1–4^ Many groups studying the T cell response to cancer have highlighted the importance of a TCF1^+^ stem-like CD8^+^ T-cell. This cell gives rise to cytotoxic daughter cells while self-renewing in chronic antigen settings.^5^ These cells have been described in several human tumor types.^6–10^ Functional experiments indicate these cells maintain proliferative capacity in the tumor and are likely the source of the anti-tumor effector.^7,8,11–13^ Our prior work found these cells reside in close proximity to densely clustered MHC-II^+^ antigen presenting cells (APC), which we termed an antigen presenting niche.^7^ In recent work, we found these TCF1^+^ cells were specific for HPV antigens in head and neck tumors, and upon restimulation with their cognate antigen, underwent proliferation and differentiated to the effector state.^8^ In many studies, the presence of this cell type in the tumor correlates with response to PD1 blockade, highlighting their importance in the immune response to cancer.^10,14,15^

While CD8^+^ T cell infiltration is prognostic for survival and TCF1^+^ CD8^+^ T cells have been found in many tumor types, it remains unclear if these observations extend to metastasis in the central nervous system. The central nervous system (CNS) has long been considered immune-privileged. Lymphocyte entry into the brain was thought to be limited by the blood brain barrier.^16,17,18^ However, several recent studies have highlighted the role of a CNS specific lymphatic system that facilitate T cell entry into the brain including into brain tumors.^19–21^ This immune infiltration of brain metastasis (BrM), specifically, has just begun to be characterized. Interestingly, the number of CD8^+^ T cells infiltrating the BrM has not always correlated with longer survival.^22–30^ Yet, BrMs respond to checkpoint immunotherapy, suggesting they can harbor a productive anti-tumor CD8^+^ T cell response.^31^ Given the complexity of T cell infiltration into BrM, this study comprehensively investigated the cellular composition and architecture of CD8^+^ T cells in these tumors. In addition, since stereotactic radiosurgery (SRS) is commonly recommended for patients with BrM with or without surgery^32,33^, we investigated how this treatment altered the CD8^+^ T cell response to cancer.

## Materials and Methods:

### Patients:

Records of patients treated at two institutions (Emory University and the Levine Cancer Institute) between 2007–2021 were evaluated and reviewed. Data were de-identified according to the Health Insurance Portability and Accountability Act, and all investigations were performed in accordance with the relevant guidelines and regulations. Informed consent was obtained for tissue sample banking; informed consent for this study was waived by the Institutional Review Board that approved the study protocol. Inclusion criteria included a pathologic diagnosis of metastatic cancer to the brain and no prior immunotherapy. Immune biomarkers evaluated by patient characteristics are show in Supplemental Table 2.

### Defining local recurrence:

Local recurrence was defined primarily based on the development of a contrast-enhancing mass within or adjacent to the prior resection cavity on MRI. If a re-resection was performed, pathologic confirmation was also utilized although not required. If there was a question of the enhancement representing local recurrence vs radiation necrosis, additional advanced imaging (eg, MR perfusion, MR spectroscopy, or brain positron emission tomography) was obtained, and consensus was reached in a multidisciplinary neuro-oncology tumor board. Additionally, the lesion was followed over time, and persistence or resolution with observation or glucocorticoids further assisted with the differentiation of local recurrence vs. radiation necrosis.

### FFPE Samples:

Formalin fixed paraffin embedded tissue samples from these patients were stained and analyzed. 67 standard of care samples were obtained from the Emory Brain Tumor Bank, and 76 pre-operative SRS samples were acquired from the Levine Cancer Institute.

### FFPE Sample preparation:

Sections were deparaffinized in successive incubations with xylene and decreasing concentrations (100, 95, 75, 50, 0%) of ethanol. Antigen retrieval was achieved using Abcam 100x TrisEDTA Antigen Retrieval Buffer (pH = 9) heated under high pressure. Sections were then washed in PBS + 0.1% Tween20 before antibody staining.

### Immunofluorescence staining:

Immunofluorescence antibody staining was done using two different techniques: (1) Sections were blocked for 30 min with 10% goat serum in 1x PBS + 0.1% Tween20. Sections were then stained with appropriate primary and secondary antibodies. Primary antibodies were incubated for 1 h at room temperature. Secondary antibodies were incubated for 30 min at room temperature. Detailed information about antibodies used are listed in Supplemental Table 4. Sections were counterstained with DAPI according to manufacturer instructions (Thermo-Fisher). (2) Immunofluorescence antibody staining was performed using the Opal 7-color immunofluorescence kit (Akoya Biosciences) endogenous peroxidase activity was quenched by microwave treatment of the slides with AR buffer. Non-specific binding was blocked with blocking/Ab diluent. After incubation with the primary antibody, the slides were incubated with HRP Ms+Rb secondary antibody and then incubated in the appropriate opal fluorophore for 10 minutes, until staining developed. The slides were finally counterstained with DAPI.

### Image capture and analysis:

The selected fluorophore panel (1) allowed for simultaneous visualization of three targets and a nuclear stain (DAPI) using a Zeiss Axio Scan.Z1 Slide Scanner equipped with a Colibri 7 Flexible Light Source. Zeiss ZenBlue software was used for post-acquisition image processing. Slides stained with the Opal IHC Kit (2) were scanned using a Perkin Elmer Vectra Polaris and allowed for simultaneous visualization of six targets and a nuclear stain. For brightfield imaging, slides were scanned using a Hamamatsu’s Nanozoomer slide scanner. Images were analysed using CellProfiler, QuPath, and custom R and python scripts, as previously described.^7^ This analysis pipeline allowed for determination of the x,y location of each cell in each image, as well as the quantitation of the distance between each cell type and the density of each cell type. Immune niches were defined as 100um × 100um cellular neighbourhoods where both TCF1+ CD8+ T cells and MHC-II+ antigen presenting cells co-localized. Proportion of tumor with immune niches was defined as the percentage of tumor tissue (percentage of 10,000um^2^ neighbourhoods) occupied by immune niches (where TCF1^+^ CD8^+^ T cells and MHC-II^+^ cells co-localize in a local 10,000um^2^ cellular neighbourhood).

### Fresh human sample collection, processing and flow staining:

BrM samples were collected after patients underwent craniotomy and surgical BrM resection. In the pSRS group, SRS was administered 1–10 days prior to craniotomy. All fresh samples (SOC and pSRS) for flow analysis were treated and acquired at Winship Cancer Institute. Samples were collected directly after resection into Phosphate Buffered Saline. The samples were then processed by getting cut into small pieces, digested with a MACS enzyme cocktail, and then homogenized using a MACS Dissociator. The digested tumor was washed then through a 70um filter to obtain a single cell suspension. Samples were then preserved in freezing media (FBS + 10% DMSO) at −80C.

Single cell suspensions from processed human tumor samples were stained with the antibodies listed Supplemental Table 5. Live/dead staining was done using fixable near-IR or aqua dead cell staining kit (Invitrogen). Cells were permed using the FOXP3 Fixation/Permeabilization kit (eBioscience) for 45 minutes with fixation/permeabilization buffer at 4C and stained with intracellular antibodies in permeabilization buffer for 30mins at 4C. Samples were acquired on a Symphony instrument and analyzed using FlowJo (v10).

### scRNA seq:

Single cell suspensions were stained and sorted on the Beckton Dickinson FACS Aria II Cell Sorter on CD45+ CD8^−^ and CD45+ CD8^+^. These two sorts were then mixed 1:1 with goal of enriching for the infiltrating CD8 T cell population. Single cell RNAseq libraries were made using the Chromium single cell 5’ Library and Gel Bead Kit (10x Genomics) and captured into the Gel Beads-in-emulsion (GEMs). After the reverse transcription GEMs were disrupted and cDNA was isolated and pooled. The barcoded cDNA was fragmented, end repair and A-tailing was done, followed by sample index PCR. The purified libraries were sequenced to 50,000 reads/cell on a HisSeq300 (Illumina) with 26 cycles for read 1, 8 cycles for index (i7) and 91 cycles for read2.

Cellranger v3.1 was used to align, filter, count the barcodes and unique molecular identifiers (UMI). Data was then analyzed using Seurat v3.0. Briefly, cells with less than 5% mitochondrial genes were used. Cells that expressed less than 200 genes or more than 2000 were excluded from analysis. Raw counts were then normalized for each UMI based on total expression, scaled by multiplying by 10,000 and then log transformed. Variable genes were determined based on average expression and dispersion, then used to perform principal component (PCA) analysis. Selected PCAs were used to generate clusters and UMAP plots. Heatmaps were generated using scaled expression data of marker genes, using the FindAllMarkers function in Seurat. Normalized gene expression data was also shown as feature plots. Gene set scoring was performed using VISION R package V2.1. Proliferation index was done as previously described.^34^ SOC had 21091 total cells; pSRS had 12597 total cells. For clonotype analysis, MiXCR was run on raw fasq for SOC2 and pSRS2. The clonotypes identified were overlaid on the UMAP of the T cell subsets.

### Statistical Analysis:

The optimal cutoff values for stem-like T cells and immune niche proportion was determined by bias-adjusted log-rank test. Local recurrence and death were regarded as two competing events. Cumulative incidence plots were created based on the proportional subdistribution hazards model and Gray’s test was performed to analyze the differences between high and low groups. SAS software version 9.4 (SAS Institute, Inc. Cary, NC) was utilized for the data analyses.

## Results:

### TCF1^+^ PD1^+^ stem-like T cells are found in brain metastases and reside in an immunological niche

**Figure 1.**
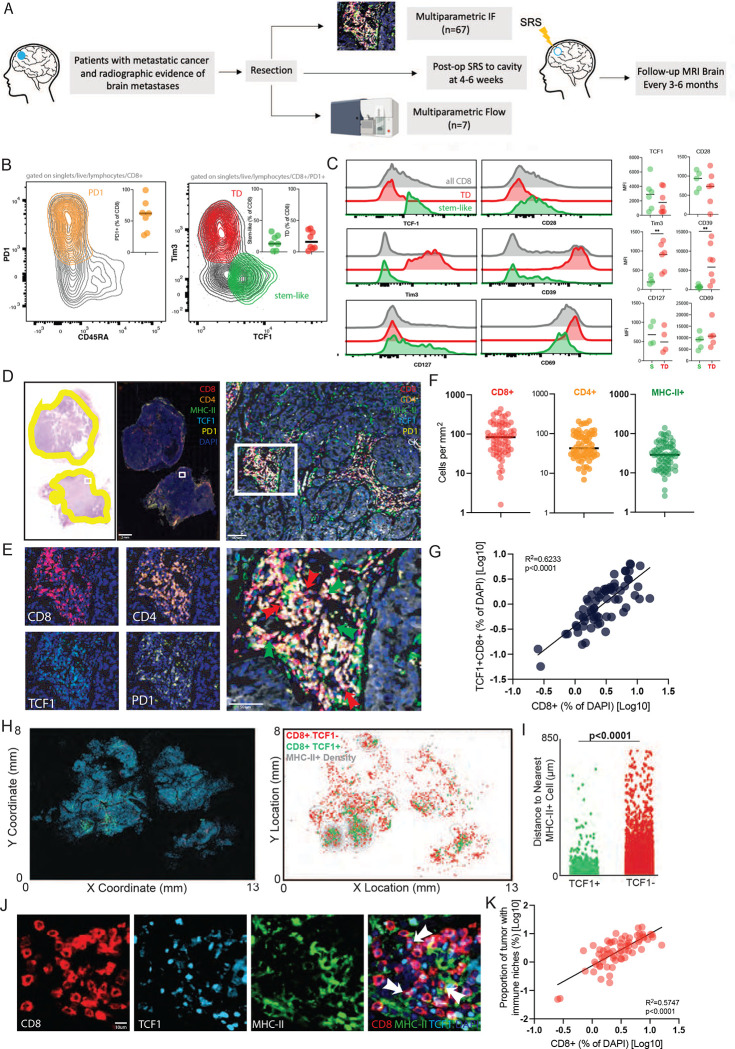
TCF1^+^ PD1^+^ stem-like T cells are found in brain metastases and reside in an immunological niche. **A)** Schema of sample collection, processing and analysis of BrM tissue. **B)** Flow cytometry characterizing PD1^+^ stem-like and terminally differentiated (TD) effector-like cells in BrM. Greater than 50% of CD8^+^ T cells in BrM are PD1^+^. **C)** Expression (mean fluorescence intensity (MFI)) of activation markers, checkpoint molecules, and transcription factors by TD and stem-like subsets, gated as in **B**. **D)** H&E with tumor regions outlined in yellow (left); immunofluorescence whole slide image (right) with region of interest shown as white box. Region of interest zoomed on immune cell cluster. **E)** Individual T cell markers, green arrows highlighting CD8^+^ PD1^+^ TCF1^+^ stem-like cells, red arrows highlighting CD8^+^ PD1^+^ TCF1^−^ terminally differentiated, effector-like cells. **F)** Quantitation of immune cell densities. **G)** Percentage of total CD8^+^ T cells correlates with percentage of TCF1^+^ CD8^+^ T cells. **H)** Cellular spatial relationship map. After acquiring x.y coordinates of MHC-II^+^ cells, MHC-II^+^ cellular density was calculated (number of MHC-II^+^ cells per 10,000 μm^2^). x,y location of CD8^+^ T cells are overlaid with MHC-II^+^ density contour. CD8^+^ cells were designated TCF1 positive or negative. **I)** Distance between CD8^+^ T cells and the closest MHC-II^+^ cell. **J)** Immunofluorescence demonstrating immune niche areas with white arrows denoting stem-like T cells. **K)** Percentage of total CD8^+^ T cells correlates with proportion of tumor tissue occupied by immune niches.

To first characterize T cells in BrM, we performed flow cytometry on freshly resected tissue from 7 patients (Figure 1A). To identify antigen reactive cells, we gated on PD1^+^ CD45RA^−^ cells. Around two thirds of the CD8^+^ cells expressed these markers, confirming their antigen reactivity (Figure 1B). Within this PD1^+^ subset, we could identify both a TCF1^+^ Tim3^−^ stem-like cell, and a TCF1^−^ Tim3^+^ cell (Figure 1B). Consistent with our observations in other human tumors outside the CNS^7,8,12^, the stem-like T cells expressed higher levels of CD28, TCF1 and CD127 and lower levels of Tim3 and CD39 compared to terminally differentiated CD8^+^ T cells (Figure 1C).

To analyze a more comprehensive cohort of patients, we collected 67 tissue samples from brain metastases resected at Winship Cancer Institute of Emory University (Figure 1A, **Supplemental Table 1**). These patients had non-small cell lung cancer (NSCLC), melanoma, or breast cancer and none had received prior immunotherapy (**Supplemental Table 1**). From these samples, we performed quantitative multiparametric immune fluorescence (IF) (Supplemental Figure 1A–E) to define the overall immune architecture of the BrM (Figure 1D, E). We found a high degree of variation between patients in the infiltration of CD8^+^ and CD4^+^ T cells as well as MHCII^+^ antigen presenting cells (APC) (Figure 1F). As with the cells analyzed by flow-cytometry, the majority of both CD8^+^ and CD4^+^ T cells were PD1^+^ indicating these cells were actively responding to antigen (Figure 1E). In these BrM, approximately 40% of the total CD8^+^ T cell population were TCF1^+^ (Supplemental Figure 1D). Of note, the frequency of CD8^+^ TCF1^+^ stem-like T cells (of DAPI^+^ cells) correlated with the frequency of total CD8^+^ T cells (R^2^ =.6233, p<0.001) demonstrating that with an increase in stem-like CD8^+^ T cells there is a concomitant overall increase in BrM CD8^+^ T cell infiltration (Figure 1G).

In prior work, we had found that TCF1^+^ CD8^+^ T cells were not randomly distributed in tumors, but instead are only found in close proximity with densely clustered APC, which we termed immune niches.^7^ Here, we found a correlation between the BrM density of stem-like T cells and MHC-II^+^ APCs (Supplemental Figure 1F) suggesting a similar inter-relationship. Next, using our digitized reconstruction of whole slide IF images, we generated contour maps of the density of MHC-II^+^ cells with green dots showing the location of TCF1^+^ CD8^+^ T cells, and red dots showing the location of TCF1^−^ T cells (Figure 1H). Stem-like T cells clearly resided in areas of higher MHC-II^+^ cell density compared to the TCF1^−^ cells (Figure 1H). Quantification of the distance of each TCF1^+^ CD8^+^ T cell to the nearest neighboring MHC-II^+^ cell found stem-like CD8 T cells were far closer on average to APCs (Figure 1I). The TCF1^−^ T cells, in contrast, were distributed throughout the tumor without any preference for the APC zones. These findings confirm the formation of an immune niche in BrM, similar to non-CNS sites (Figure 1J). Notably, prior data suggests that the niche is important for supporting and maintaining the CD8^+^ T cell response within non-CNS tumors.^12^ Similarly, here we demonstrate a correlation between immune niche density and the frequency of total CD8^+^ T cell infiltration, again suggesting that these niches have a supportive role in BrM (Figure 1K).^6^ Overall, these data indicate the metastasis to the CNS are infiltrated by distinct functional subsets of CD8^+^ T cells: a TCF1^+^ stem-like population, and a TCF1^−^ effector-like population. Furthermore, these data indicate that although these metastases have entered an immunological environment where T cell access can be limited, a T cell response is successfully mounted that is consistent with other solid tumor types.

### High immune niche density is associated with longer BrM local control

Local BrM recurrences are a complex problem often with limited treatment options.^35^ Therefore, a prognostic biomarker which could identify specific patients at highest risk for local recurrence would allow for early treatment escalation. In other tumor types outside the CNS, we previously showed that both a high density of total CD8^+^ T cells and the immune niche were associated with longer progression free survival.^7^ We, therefore, investigated whether these were similarly prognostic for local control in BrM. First, we assessed the degree of CD8^+^ T cell variability across all 67 samples and found substantial differences between BrM (Figure 1F). Next, we evaluated whether high CD8^+^ T cell density was associated with longer local control using a competing risk analysis. This approach allowed us to account for the competing events of death and local recurrence. In contrast to tumors outside the CNS, high CD8^+^ T cell density was not associated with longer local control (Supplemental Figure 2A). This suggests this one general cell type may not fully capture the complex interplay of different immune cells involved in BrM control.

**Figure 2.**
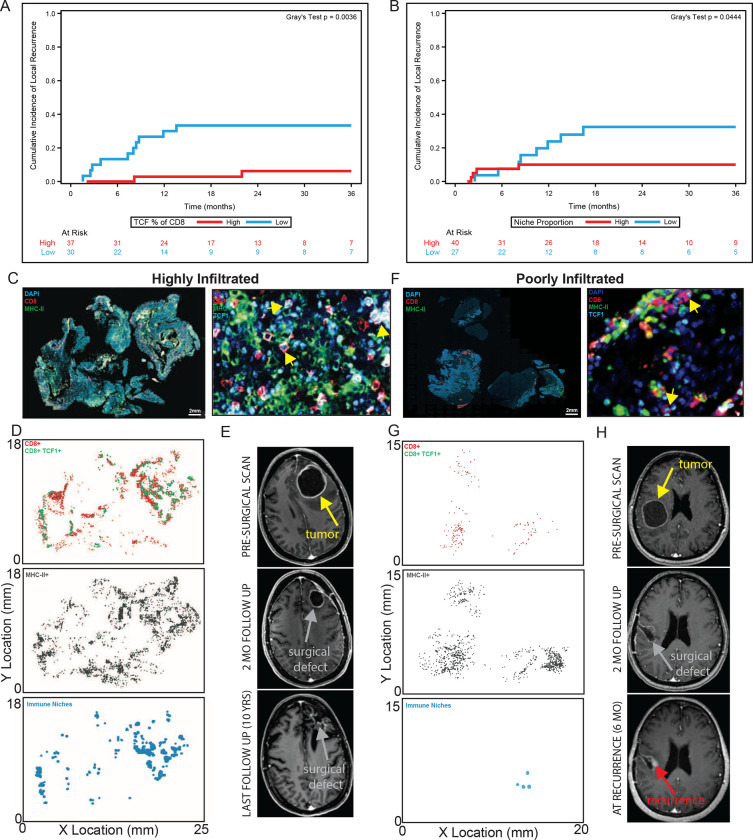
Higher immune niche density is associated with longer local control. **A)** BrMs with a higher percentage of TCF1^+^ of CD8^+^ T cells had extended local control of disease. Gray’s test, p=0.0036. **B)** BrMs with a higher tumor niche proportion had extended local control of disease. Gray’s test, p=0.0444. **C)** Overview (left) and region of interest (right) immunofluorescence for a highly immune infiltrated tumor. Yellow arrows denote stem-like T cells. **D)** x,y location maps of CD8^+^ T cells, MHC-II^+^ cells and immune niches. **E)** Cystic brain metastasis pre-operative and post-operative resection cavity followed for 10-years without evidence of local recurrence. **F)** Overview (left) and region of interest (right) immunofluorescence of poorly infiltrated tumor. Yellow arrows denote stem-like T cells. **G)** x,y location maps as in (D). **H)** Nodular local recurrence at surgical cavity margin occurs at 6 months post-operatively denoted by red arrow.

Next, we turned our attention to the immune niche and also found significant variability in the niche density across BrMs (Supplemental Figure 2B). In contrast to bulk CD8^+^ T cells, BrM with a higher frequency of stem-like T cells or with a higher density of immune niches had longer local control (Figure 2A, B). In a representative highly infiltrated BrM, there were many areas of high T cell density and high APC density, and importantly, many areas where stem-like T cells and APCs co-localized, forming immune niche clusters that were distributed throughout the BrM (Figure 2C, D). Following treatment, the resection cavity remained free of local recurrence at 10 years (Figure 2E). In contrast, a representative poorly infiltrated BrM (Figure 2F) showed evidence of local recurrence 6 months from the end of treatment, lacked a significant density of immune infiltration, and importantly, lacked the widespread presence of these intratumoral immune niches (Figure 2G, H). The immune niche, therefore, has potential as a prognostic biomarker as it captures a link between the immune microenvironment and patient outcomes which is not revealed by bulk CD8^+^ T cell analysis. More specifically, the immune niche may have an active role in restraining tumor growth/recurrence by maintaining an on-going anti-tumor CD8^+^ T cell response.

### The impact of pre-operative SRS on the immune niche

**Figure 3.**
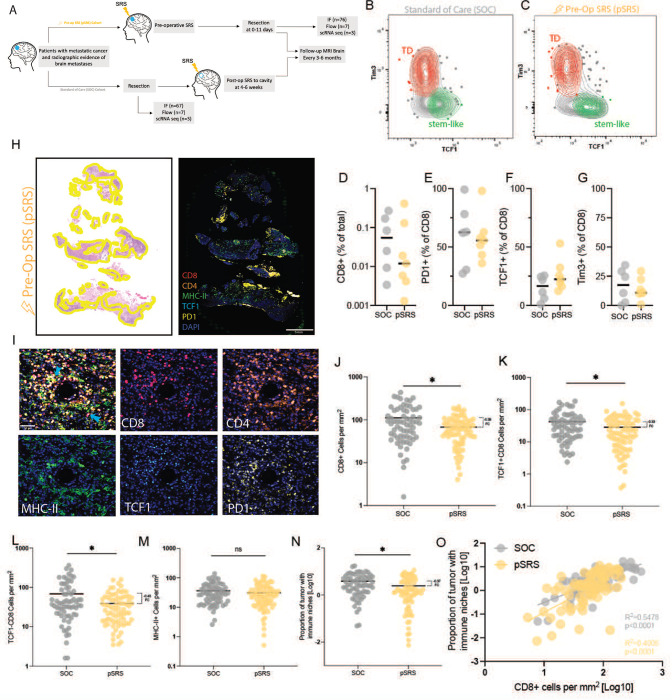
The impact of pre-operative SRS on the immune niche. **A)** Schema of sample collection, processing, and analysis of BrM tissue including pre-operative SRS (pSRS) specimens. **B)** Flow cytometry demonstrating the presence of TD and stem-like T cells in both upfront resected (standard of care, SOC) and **C)** pSRS BrM. **D)** Percent CD8^+^ T cells of total cells in BrM SOC vs pSRS, p= 0.8904. **E)** Percent PD1^+^ of CD8^+^ T cells in BrM SOC vs pSRS, p= 0.8708. **F)** Percent TCF1^+^ of CD8^+^ T cells in BrM in SOC vs pSRS, p= 0.1400. **G)** Percent Tim3^+^ of CD8^+^ T cells in BrM in SOC vs pSRS, p= 0.6655. In D-G, p values calculated by unpaired t test. **H)** H&E with tumor regions outlined in yellow (left) and immunofluorescence whole slide image (right). **I)** Merged image and individual stains for T cell subsets and MHC-II^+^ cells. **J)** Total CD8^+^ cells per mm^2^ are lower in pSRS samples compared to SOC, p= 0.0006. **K)** TCF1^+^ are also lower in pSRS samples, p= 0.0175. **L)** TCF1^−^ effector-like are lower in pSRS vs SOC, p= 0.0023. **M)** There is no difference in MHC-II^+^ cell per mm^2^ in SOC vs pSRS, p= 0.2647. **N)** Proportion of tumor with immune niche is lower in pSRS compared to SOC likely due to the decrease in stem-like T cells, p= 0.0058. In J-N, *:p<0.05, as calculated by unpaired t test. **O)** Proportion of tumor with immune niche correlates with CD8^+^ cells per mm^2^ in both pSRS and SOC BrM.

Current standard of care (SOC) for BrM is upfront surgical resection followed by post-operative SRS or SRS alone. In our preclinical studies, focal radiation increases stem-like CD8^+^ T cell infiltration into tumor tissue, which led us to investigate the impact of preoperative stereotactic radiosurgery (pSRS) on the T cell populations and the immune niche in BrM (Figure 3A).^36^ Importantly, our group has been a pioneer in the pSRS and has described several potential clinical benefits of such a practice.^37^ The ability to optimally sequence these treatment modalities is notable given the known immune-stimulatory activity of radiation.^38^ Currently, it is unknown whether pSRS has similar immunostimulatory activity in the brain and what impact it may have on the immune niche.

A total of 76 patients who received pSRS were analyzed by quantitative IF and 7 patients who received pSRS were analyzed by flow cytometry. These BrM were primarily lung and melanoma. The median time from pSRS to surgery was 2 days with a median dose of 15 Gy (**Supplemental Table 3**). The pSRS and SOC cohort patient characteristics were, overall, very similar (**Supplemental Table 4**).

In the pSRS BrM, we again identified PD1^+^ stem-like and terminally differentiated CD8^+^ T cells by flow cytometry (Figure 3B, C). The terminally differentiated and stem-like T cells following pSRS appeared phenotypically similar to SOC, with terminally differentiated cells continuing to express robust levels of Tim3, CD39 and lower levels of TCF1 and CD28 (Supplemental Figure 3A, B). Quantitative flow cytometry analysis of pSRS BrM, demonstrated a strong trend towards a decrease in the overall CD8^+^ T cell frequency with no change in the frequency of PD1^+^ CD8^+^ T cells (Figure 3D, E). Additionally, no significant changes were observed in the frequencies of stem-like or terminally differentiated subsets (Figure 3F, G).

In our larger cohort analyzed by quantitative IF, similar to SOC BrM, we could identify TCF1^+^ stem-like CD8 T cells in the tumor tissue (Figure 3 H, I) and identified a strong correlation between CD8^+^ T cell and stem-like CD8^+^ T cell frequencies (Supplemental Figure 3C). Consistent with our flow data, a lower density of CD8^+^ T cells was observed in the pSRS vs SOC cohort (Figure 3J). Evaluating the individual subsets, we found a small, but significant difference in density of the TCF1^+^ T cells population as well as a larger magnitude difference in the TCF1^−^ cell population (Figure 3K, L). There was, however, no difference in the MHC-II^+^ density between SOC and pSRS (Figure 3M). Finally, while niche density was slightly attenuated following pSRS compared to SOC BrM controls (Figure 3N), the correlation between niche proportion and CD8^+^ T cell density was maintained (Figure 3O). These data suggest that while pSRS may have differential effects on T cell subsets, MHC-II^+^ myeloid cells may be less affected and, importantly, intratumoral immune niche organization is maintained.

### The transitory CD8^+^ T cell population is preferentially reduced following pSRS

**Figure 4.**
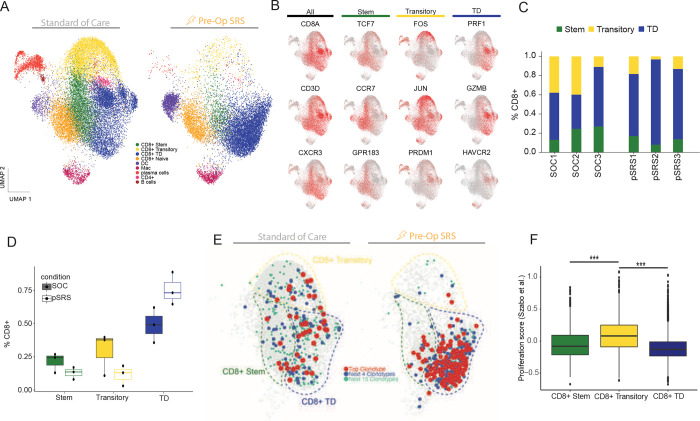
The transitory CD8^+^ T cell population is preferentially reduced following pSRS. **A)** Single cell RNA sequencing of SOC and pSRS BrM. Multiple different immune populations were identified under both treatment conditions. Stem-like and terminally differentiated effector-like T cells were observed as well as a transitory subset. **B)** Feature plots demonstrating expression of stem, transitory, and terminally differentiated effector-like cell markers. **C)** T cell subset frequency in three different SOC and three pSRS BrM. **D)** Frequency of each subset under both conditions. pSRS demonstrates a strong trend towards decreased transitory cell frequency and increased terminally differentiated effector-like frequency. **E)** Clonotypic analysis demonstrating CDR3 TCR overlap between all 3-antigen experienced CD8^+^ T cell subsets. **F)** Proliferation score showing increased proliferation in the transitory subset relative to the stem-like and terminally differentiated effector-like T cells. ***: p<0.001 by Mann Whitney.

To more specifically determine the impact of pSRS on T cell subsets, we evaluated the CD8^+^ and other immune cell’s transcriptional signature by sorting CD45^+^ cells from 3 SOC and 3 pSRS BrM and performed single cell RNA sequencing. In the SOC BrM we found, consistent with prior reports, an array of different immune cells including dendritic cells (DC), macrophages, plasma cells, B cells and CD4^+^ T cells (Figure 4A, Supplemental Figure 4D).^25,26^ Within the CD8^+^ T cell population, we found three antigen experienced clusters and one naïve cell cluster, as previously described. (Figure 4A).^8^ The first antigen experienced cluster, Cluster 1, expressed CXCR3, TCF7, CCR7 and GPR183 markers consistent with the stem-like subset (Figure 4B). This stem-like cluster also expressed low levels of effector molecules including GZMB and PRF1. Cluster 2 expressed high levels of FOS, JUN and lower levels of both effector molecules GZMB, PRF1 and stem-like molecules TCF7, CCR7 consistent with a transitory phenotype between Cluster 1 and 3, analogous to those seen in other tumor types.^8,39^ Cluster 3 (the terminally differentiated effector-like cluster) was characterized by high PRF1, GZMB, and HAVCR3 expression and low expression of the stem-like markers (Figure 4B). Clonotypic analysis confirmed significant TCR overlap between these 3 clusters further supporting a previously described lineage relationship (Figure 4E).^8^ In the pSRS BrMs, all 3-antigen experienced CD8^+^ T cell clusters were also identified (Figure 4A, C). Notably, the transitory cluster was found at a much lower frequency in the pSRS than in the SOC BrMs (Figure 4C, D). The terminally differentiated subset, in contrast, demonstrated increased frequency in the pSRS compared to SOC BrMs (Figure 4D). The immunodominant TCR clone was also enriched in the terminally differentiated cluster in the pSRS compared to SOC (Figure 4E).

Cells actively dividing are known to be the most radiosensitive^40^, and preclinical results have shown the transitory cells to be the most proliferative of the 3 subsets.^39^ Here, we also found that the transitory subset had a higher proliferation score than either the stem-like T cells or terminally differentiated effectors which likely accounts for their preferentially reduced frequency in the pSRS cohort (Figure 4F). We also compared the stem-like, transitory, and terminally differentiated populations between the SOC and pSRS and found notable transcriptional changes (Supplemental Figure 4A). In the stem-like population following pSRS, TXNIP was significantly upregulated. TXNIP is associated with the response to reactive oxygen species, suggesting this may be a mechanism for stem-like T cell survival after exposure to the pSRS insult.^41^ Further investigation is needed.

Next, we examined the other infiltrating immune cells and found a trend towards an increase in the frequency of both DC and macrophages following pSRS (Supplemental Figure 4B). This finding is likely due to their relative radioresistance and ability to withstand the cellular stress imparted by exposure to radiation therapy.^42^ Notably, we found that pSRS promotes a Type I interferon (IFN) response phenotype in these two APC types (DCs and macrophages), with significant upregulation of both interferon regulatory factor 5 (IRF5) and IRF8 (Supplemental Figure 4C). The Type 1 IFN response is associated with maturation of DCs, and appropriately provided co-stimulation is known to be critical for stem-like T cell activation and acquisition of effector function.^43^ In summary, pSRS has a broad impact on a diverse array of different cells. Its specific effect on CD8^+^ T cells is subtype dependent with the most potent depletion seen in the transitory population, followed by a more modest impact on the stem-like subset while the terminally differentiated effector-like cells were the least affected. ^43,44^

### Temporal changes of BrM immune niche components following pSRS

**Figure 5.**
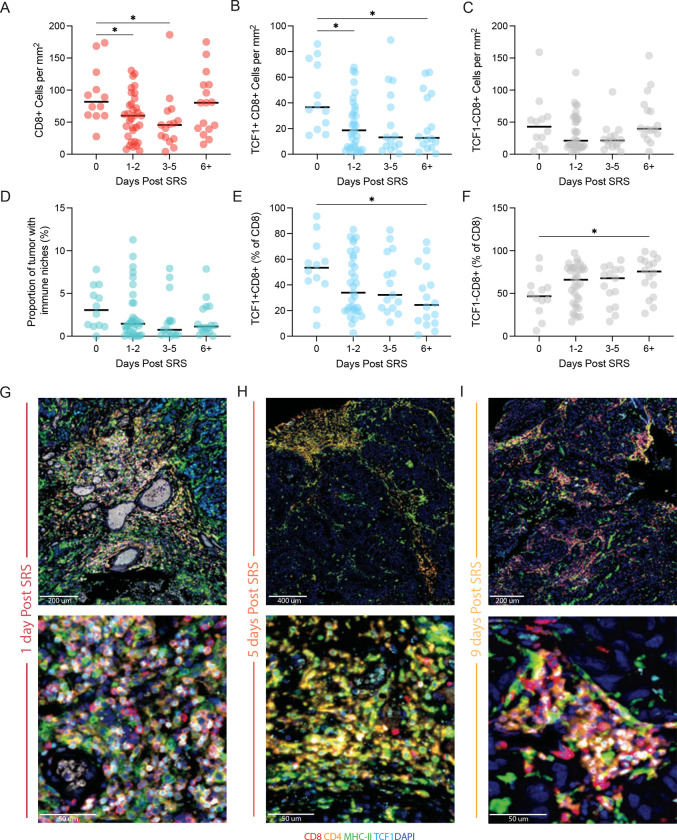
Temporal changes of BrM immune niche components following pSRS. **A)** Number of CD8^+^ cells per mm^2^ decreases from day 0 to day 5 and then rebounds to baseline by day 6+ following pSRS. **B)** Number of TCF1^+^ CD8^+^ cells decrease following pSRS and remain lower at day 6+. **C)** Number of TCF1^−^ CD8^+^ cells have a numeric, but not significant decrease from day 0–5 with a rebound by day 6+. **D)** Proportion of tumor occupied by immune niches showed a numeric but not significant decrease from day 0 to day 6+. **E)** Percentage of TCF1^+^ CD8^+^ cells significantly decreased from day 0 to day 6+. **F)** In contrast, the percentage of TCF1^−^ CD8^+^ cells significantly increased from day 0 to day 6+. In A-F, *:p < 0.05 as calculated by ordinary one-way ANOVA. **G)** Representative immunofluorescence of the immune niches and T cells subsets at day 1, **H)** at day 5, **I)** and at day 9 after pSRS.

To further investigate the relative persistence of the terminally differentiated effector-like cells following pSRS, we performed a kinetic analysis evaluating the changes in pSRS treated BrM. In patients who had BrM resected on the same day as the pSRS, there was no significant difference in the number of total CD8^+^, TCF1^+^ stem-like or TCF1^−^ cells in the tumor compared to SOC (Supplemental Figure 5A–C). In comparison, tissue resected between 1 and 5 days post pSRS had significantly lower numbers of total, TCF1^+^ stem-like, and TCF1^−^ terminally differentiated CD8^+^ T cells (Figure 5A–C). However, after 6 or more days, while the TCF1^+^ stem-like population remained depressed, the TCF^−^ terminally differentiated cells had returned to baseline levels. Over the treatment time course, there was no change in the numbers of MHC-II^+^ cells (Supplemental Figure 5D). However, consistent with the reduction in the stem-like T cell population, there was a trend towards a decrease in the immune niche density in the BrM over the time course, and this had not recovered by the 6+ timepoint (Figure 5D). Representative images of tumors showing niche morphology at illustrative time points are shown in Figure 5G–I.

Together with the scRNAseq data in Figure 4, these data suggest that there is a broad depletion of T cells following radiation. The relatively quiescent TCF1^+^ stem-like cells have not yet recovered even by day 6 but given the extensive data in pre-clinical models suggesting these intra-tumoral stem-like cells are the source of effector-like cells in the tumor, we conclude that the recovery in the terminally differentiated cells seen at D6+ following pSRS is likely driven by these remaining stem-like cells. Overall, these data indicate that while pSRS does have an impact on intra-tumoral T cell populations, they remain functionally able to generate an anti-tumor immune response.

## Discussion:

In this study—the largest such study to our knowledge evaluating BrM immune architecture—we sought to understand the tumor immune microenvironment of BrM following up-front tumor resection (SOC) or pre-operative SRS. We found that immune niches (similar to those previously described in primary renal cell carcinoma, consisting of antigen presenting cells and stem-like T cells) were present at varying densities in BrM under both treatment conditions (Figure 1, 3).^7^ Remarkably, similar to tumors outside the CNS, high BrM niche density was associated with longer local control of disease (Figure 2). In the pSRS cohort, there was a reduction in total BrM CD8^+^ T cells and both the TCF1^+^ and TCF1^−^ subsets relative to SOC (Figure 3). Notably, the immune niche organization was maintained following pSRS (Figure 3). By scRNA seq, pSRS most potently depleted the proliferating transitory population while the terminal effectors were less affected (Figure 4). Finally, when the pSRS BrMs were evaluated by time of resection after pSRS, the total CD8^+^ T cell population demonstrated a rebound by day 6+ driven by an increase in the frequency of the TCF^−^ CD8^+^ T cells (Figure 5).

Taken together, these data fit into and build upon an expanding body of work evaluating the tumor-microenvironment in BrM.^22–26^ Importantly, our study is hypothesis-generating with a few limitations. Due to the sample size, a multivariate analysis for niche density and local control was not possible. However, a local control end point was selected as it has fewer confounders than distant brain failure, which is impacted by systemic disease burden, or overall survival, which has innumerable confounders. Future expanded analyses will evaluate these other important endpoints in detail. Of note, the parallel results between metastatic (reported here) and primary sites of different histologies reinforces the validity and importance of the link between higher niche density and improved cancer control.^7^ Validation of this biomarker in an independent cohort, as well as further expanding the number and diversity of histologies of the BrM evaluated is the focus of our ongoing studies. Additionally, given the focus of this study was the CD8^+^ T cell response, a deeper analysis of APC subtypes in the immune niche is planned. Here, by single cell RNA seq, we confirmed there are macrophages, dendritic cells, and B cells in the BrM, as shown by other investigators.^23,24^

The pre-operative SRS findings reported here were highly novel and intriguing to us. We had hypothesized, based on our preclinical studies, pre-operative SRS would increase the density of stem-like T cells and the presence of immune niches around day 7 following radiation.^36^ However, in our kinetics analysis in Figure 5, we did not find an increase above baseline by day 6+, but we did find a rebound in total CD8^+^ T cell numbers that appeared to be driven by an increase in the frequency of the terminally differentiated subset. These results suggest that SRS, like anti-PD1 therapy, may drive both the proliferation and differentiation of stem-like T cells into terminally differentiated, effector-like cells.^5,13^ This mechanism would account for the rebound in total CD8^+^ T cells and the relative frequency changes in stem-like and terminally differentiated, effector-like cells. Importantly, the newly generated effectors following pSRS may be superior to the baseline TCF^−^ effectors at controlling disease suggesting that the rebound at day 6+ may confer a clinical benefit beyond a return to baseline CD8^+^ T cell numbers.^45^ Further *in vivo* and *in vitro* functional studies are necessary to confirm this hypothesis.

The larger significance of this clinically important study should not be understated. Broadly, these findings demonstrate that despite the brain having several barriers to T cell infiltration, an immune response is capable of being mounted that resembles that of many other tumor types/locations and benefits patient outcomes. Clinically, these data suggest that the immune niche may not only be an important prognostic factor for outcomes, but also be predictive of an intracranial response to immunotherapy given the known importance of both stem-like CD8^+^ T cells and co-stimulation for a robust response to anti-PD1 therapy.^5,13,43^ These results also provide novel insight into the optimal timing of surgical resection following SRS and integrating SRS with immunotherapy. Our data indicates that resection of BrM <6+ days following pre-operative SRS may limit the immunostimulatory benefit of SRS and potentially reduce the local control benefits due to the acute decline in CD8^+^ T cells. Additionally, administering anti-PD1 therapy at the T cell nadir likely also blunts the potential synergy of combinatory SRS and anti-PD1 therapy. These data can, therefore, be used immediately to help guide intracranial BrM management and inform future clinical investigation into optimal sequencing and combination of multiple therapeutic modalities. Future clinical studies are needed to further elucidate the immune-stimulatory potential of SRS and whether an intra-cranial abscopal response can be generated.

## Supplementary Material

Supplement 1

## Figures and Tables

**Table 1:** Descriptive Statistics - Standard of Care

Variable	Level	N (%) = 67

ECOG	<2	55 (82.1)
	>=2	12 (17.9)
Sex	Male	25 (37.3)
	Female	42 (62.7)
Primary Site	Lung	42 (62.7)
	Breast	16 (23.9)
	Melanoma	9 (13.4)
Age	Mean	58.61
	Median	58.00
	Minimum	32.00
	Maximum	83.00
	Std Dev	11.62
	Missing	0.00
Dexamethasone Dose (mg/day)	Mean	15.95
	Median	16.00
	Minimum	4.00
	Maximum	40.00
	Std Dev	6.99
	Missing	27.00

**Table 2 -:** Univariate Associations

Univariate Association with CD8 cells per mm2				
			**CD8/mm2**	
**Variable**	**Level**	**N**	**Median**	**Kruskal-Wallis P-value**

ECOG	<2	55	87.48	0.503
	>=2	12	78.74	
Gender	Male	25	87.48	0.866
	Female	42	82.26	
Primary Site	Lung	42	81.85	0.506
	Breast	16	73.50	
	Melanoma	9	106.25	


		**CD8/mm2**	
**Variable**	**N**	**Spearman CC**	**Spearman P-value**

Age	67	0.032	0.797
Steroid Dose (mg/day)	40	0.220	0.173


Univariate Association with TCF percentage of CD8				
			**TCF % of CD8**	
**Variable**	**Level**	**N**	**Median**	**Kruskal-Wallis P-value**

ECOG	<2	55	39.31	0.719
	>=2	12	43.08	
Gender	Male	25	36.23	0.509
	Female	42	40.78	
Primary Site	Lung	42	36.36	0.198
	Breast	16	38.42	
	Melanoma	9	53.01	


		**TCF % of CD8**	
**Variable**	**N**	**Spearman CC**	**Spearman P-value**

Age	67	0.150	0.224
Steroid Dose (mg/day)	40	0.019	0.906


Univariate Association with Niche Proportion				
			**Niche Proportion**	
**Variable**	**Level**	**N**	**Median**	**Kruskal-Wallis P-value**

ECOG	<2	55	2.63	0.794
	>=2	12	2.96	
Gender	Male	25	3.60	0.364
	Female	42	2.45	
Primary Site	Lung	42	2.68	0.134
	Breast	16	1.53	
	Melanoma	9	5.57	


		**Niche Proportion**	
**Variable**	**N**	**Spearman CC**	**Spearman P-value**

Age	67	0.243	0.113
Steroid Dose (mg/day)	40	0.132	0.416

**Table 3:** Descriptive Statistics - pSRS

Variable	Level	N (%) = 76

ECOG	<2	59 (77.6)
	>=2	17 (22.4)
Sex	Male	40 (52.6)
	Female	36 (47.4)
Primary Site	Lung	46 (60.5)
	Breast	4 (5.3)
	Melanoma	15 (19.7)
	GI	5 (6.6)
	Other	6 (7.9)
Age	Mean	61.06
	Median	59.77
	Minimum	26.47
	Maximum	95.30
	Std Dev	12.13
	Missing	0.00
Dexamethasone Dose (mg/day)	Mean	11.60
	Median	12.00
	Minimum	0.00
	Maximum	24.00
	Std Dev	4.85
	Missing	26.00
Days from pre-op SRS to surgery	Mean	3.03
	Median	2.00
	Minimum	0.00
	Maximum	11.00
	Std Dev	2.94
	Missing	0.00
Preop SRS dose	Mean	15.86
	Median	15.00
	Minimum	12.00
	Maximum	30.00
	Std Dev	2.88
	Missing	0.00

**Table 4:** Descriptive Statistics by Group

			Group		
Covariate	Statistics	Level	SOC N=67	pSRS N=76	P-value*

ECOG	N (Col %)	<2	55 (82.09)	59 (77.63)	0.508
	N (Col %)	>=2	12 (17.91)	17 (22.37)	
Sex	N (Col %)	Male	25 (37.31)	40 (52.63)	0.066
	N (Col %)	Female	42 (62.69)	36 (47.37)	
Age	N		67	76	0.221
	Mean		58.61	61.06	
	Median		58	59.77	
	Min		32	26.47	
	Max		83	95.3	
	Std Dev		11.62	12.13	
Dexamethasone Dose (mg/day)	N		40	50	**0.002**
	Mean		15.95	11.6	
	Median		16	12	
	Min		4	0	
	Max		40	24	
	Std Dev		6.99	4.85	

* The p-value is calculated by ANOVA for numerical covariates; and chi-square test or Fisher’s exact for categorical covariates, where appropriate.

**Table 5:** Antibodies

Target Antibody	Type	Clone	Concentration	Fluorophore
CD8	Mouse IgG1, k	C8/144B	1:100	Opal 570
CD4	Rabbit		1:300	Opal 690
TCF1	Rabbit	C69D9	1:200	Opal 520
MHC-II	Mouse IgG2a, k	Tu39	1:75	Opal 620
PD1	Mouse IgG2a, k	EH33	1:100	Opal 480
Cytokeratin	Mouse IgG1, k	AE1/AE3	1:500	Opal 780
CD4	Mouse IgG2b, k	OKT4	1:100	BUV496
CD8	Mouse IgG1, k	RPA-T8	1:100	BUV661
PD1	Mouse IgG1, k	EH12.1	1:100	BUV737
CD39	Mouse IgG1, k	A1	1:100	BV421
CD45RA	Mouse IgG2b, k	HI100	1:100	BV510
CD3	Mouse IgG1, k	UCHT1	1:100	PerCP-Cy5.5
Tim3	Rat IgG2a	344823	1:50	PE
CD28	Mouse IgG1, k	CD28.2	1:100	BUV395
CD127	Mouse IgG1, k	A019D5	1:100	PE-Cy7
CCR7	Mouse IgG2a, k	G043H7	1:100	BV785
HLA-DR	Mouse IgG2a, k	L243	1:100	BV605
Tox	Rat IgG2a, k	TXR10	1:100	eFluor 660
GranzymeB	Mouse IgG1, k	GB11	1:100	A700
TCF1	Rabbit		1:100	AF488
Ki67	Mouse IgG1, k	Ki-67	1:50	BV711
CD69	Mouse IgG1, k	FN50	1:100	BV650
Foxp3	Mouse IgG1, k	206D	1:100	PE/Dazzle594
