# Supplementary material for: Immune niches in brain metastases contain TCF1+ stem-like T cells, are associated with disease control and are modulated by preoperative SRS
Source: Res Sq. 2023 Mar 23:rs.3.rs-2722744. Preprint. [Version 1] doi: 10.21203/rs.3.rs-2722744/v1 (PMC10055679; doi:10.21203/rs.3.rs-2722744/v1)
Supplement: Supplement 1 [file NIHPPrs2722744v1-supplement-1.pdf]

Supplemental Figure 1

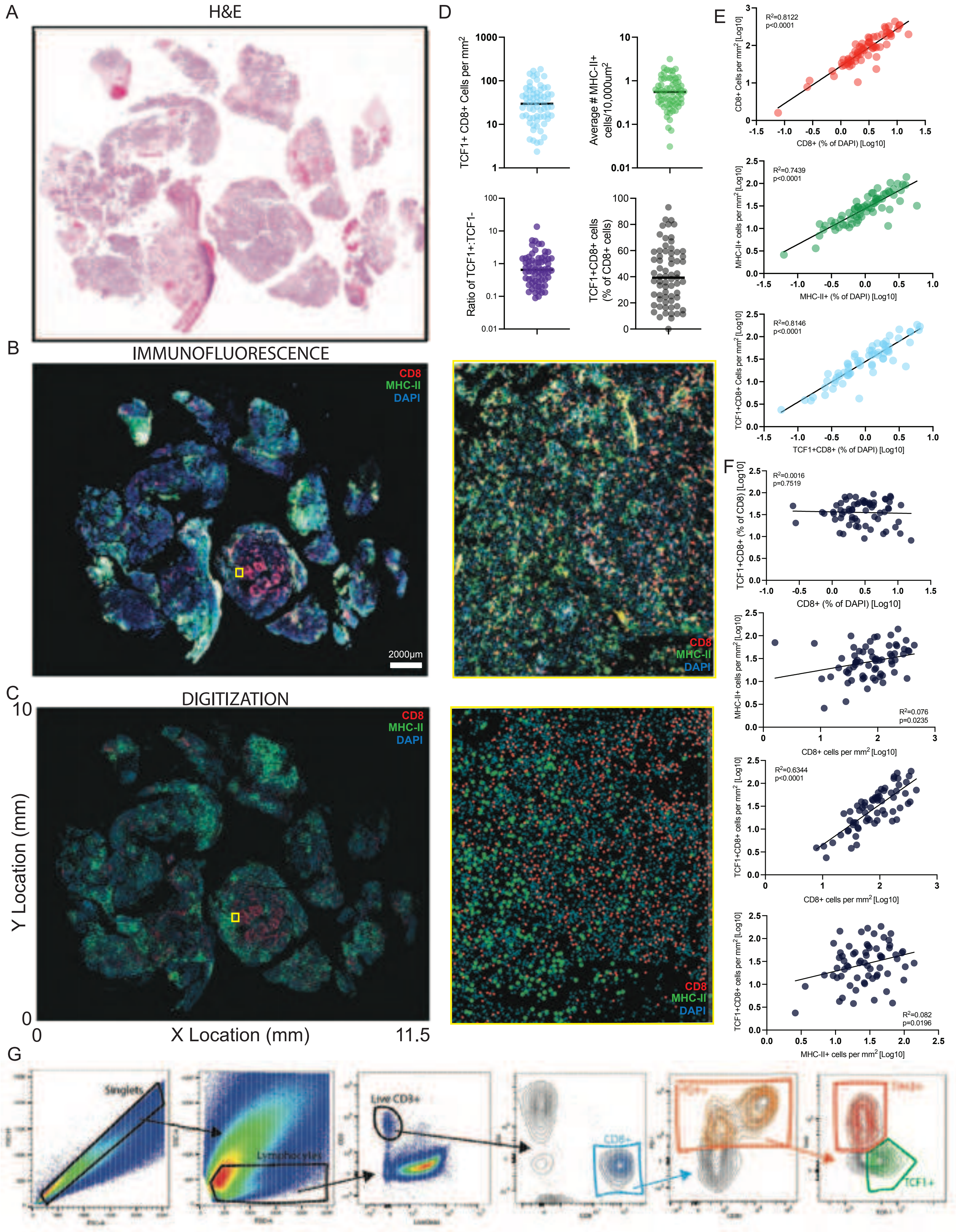

Supplemental Figure 1. A) Whole slide H&E image of BrM. **B)** Representative three-color immunofluorescence staining with region of interest shown in yellow box with region of interest on the right. **C)** Digitization of the slide with region of interest shown with region of interest on the right. **D)** Quantification of number of $CD8^+$ $TCF1^+$ cells per mm^2, average number of MHC-II^+ cells per 10,000um^2, ratio of $TCF1^+$ to $TCF1^-$ cells, and percentage of $TCF1^+$ of $CD8^+$ cells. **E)** Validation of quantification methods, demonstrating correlation between a given cell count per mm^2 and respective proportion of total cells. **F**) Correlation between percent $CD8^+$ of total cells and percent $TCF1^+$ of $CD8^+$ T cells, correlation between $CD8^+$ cells per mm^2 and MHC-II^+ cells per mm^2, correlation between CD8 cells per mm^2 and $TCF1^+$ $CD8^+$ cells per mm^2, correlation between MHC-II^+ cells per mm^2 and $TCF1^+$ $CD8^+$ cells per mm^2. **G)** Flow cytometry gating strategy.

Supplemental Figure 2

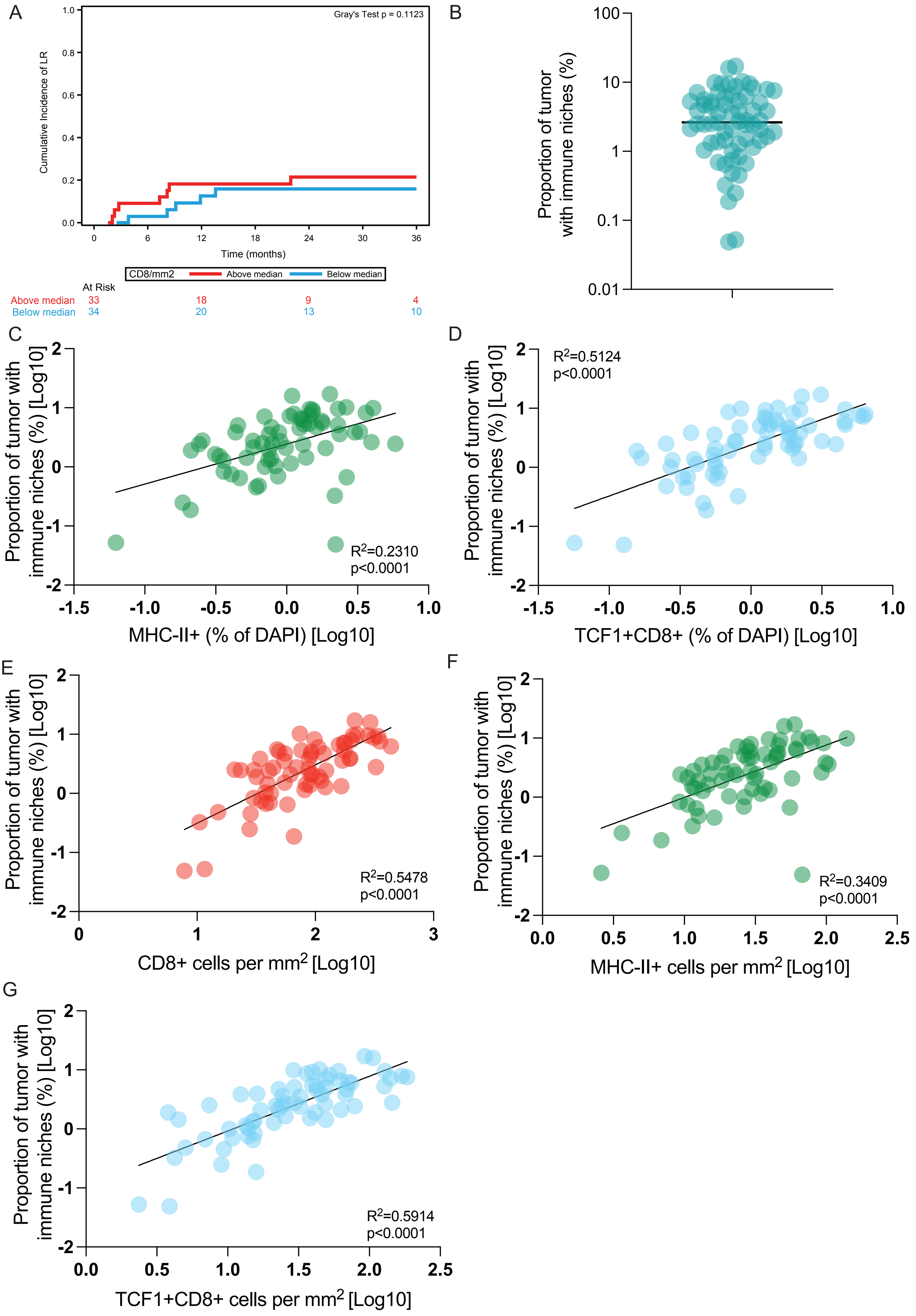

Supplemental Figure 2. A) No difference in local control between high vs low $CD8^+$ T cell per mm^2. **B)** Quantification of the proportion of tumors occupied by immune niches (local 100um x 100um cellular neighborhoods where $TCF1^+$ $CD8^+$ T cells and MHC-II^+ cells co-localize). **C)** Correlation between MHC-II^+ cell frequency and proportion of tumors with immune niches. **D)** Correlation between $TCF1^+$ $CD8^+$ cell frequency and proportion of tumors with immune niches. **E)** Correlation between $CD8^+$ cells per mm^2 and proportion of tumors with immune niches. **F)** Correlation between MHC-II^+ cells per mm^2 and proportion of tumors with immune niches. **G)** Correlation between $TCF1^+$ $CD8^+$ cells per mm^2 and proportion of tumors with immune niches.

Supplemental Figure 3

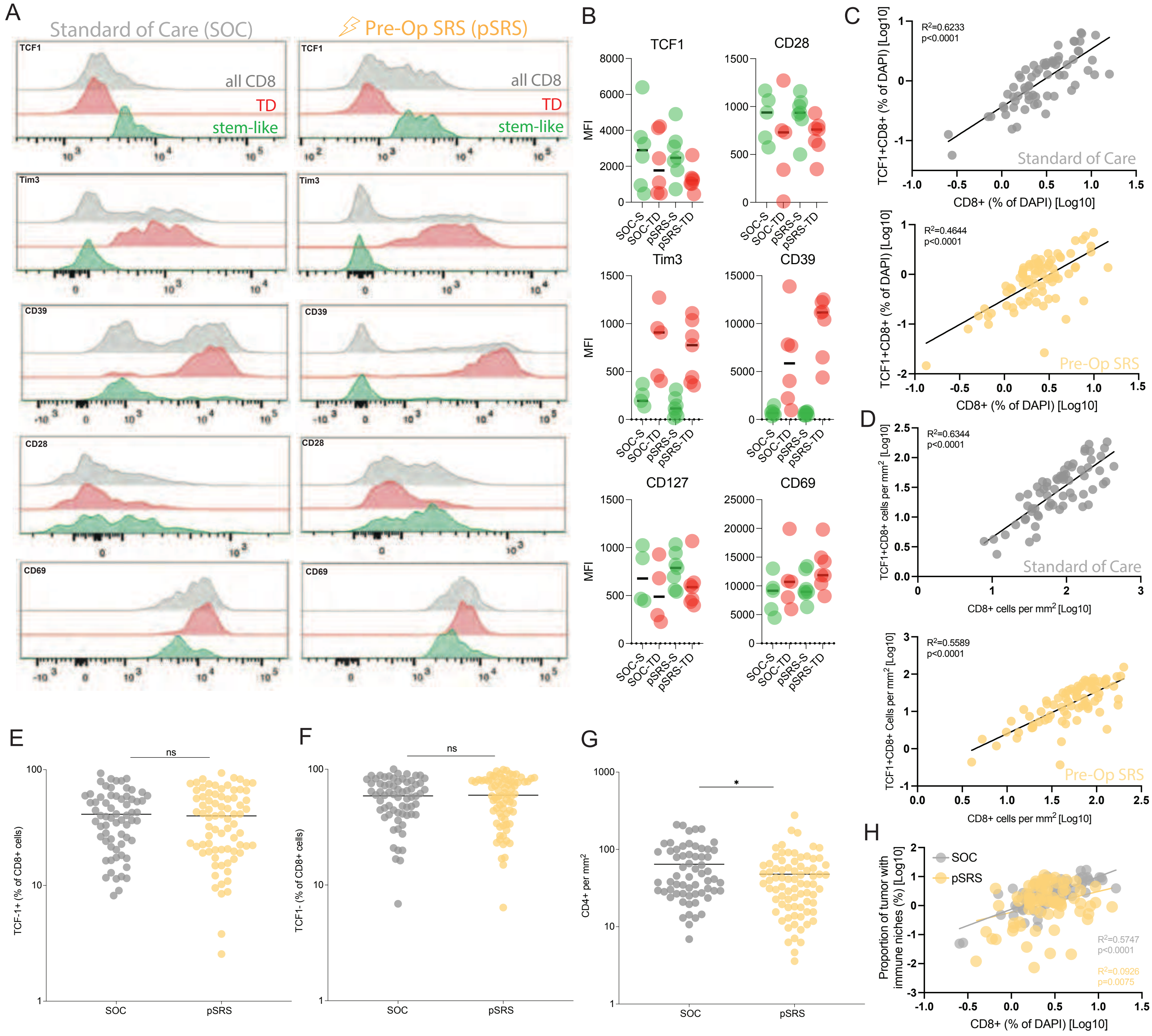

Supplemental Figure 3. A) Representative expression of stem-like and terminally differentiated effector-like cell markers in T cells from SOC and pSRS BrM by flow cytometry. **B)** Quantification of marker from (A). **C)** Correlation between frequency of $CD8^+$ cells and $TCF1^+$ $CD8^+$ cells in SOC and pSRS BrM. **D)** Correlation between numbers of $CD8^+$ cells per mm^2 and $TCF1^+$ $CD8^+$ cells per mm^2 in SOC and pSRS BrM. **E)** Concordance between frequency of $TCF1^+$ of $CD8^+$ cells in SOC and pSRS BrM, p= 0.9367. **F**) Concordance between frequency of $TCF1^-$ of $CD8^+$ cells in SOC and pSRS BrM, p= 0.8329. **G)** A significant decrease seen in $CD4^+$ cells per mm^2 in pSRS compared to SOC BrM, p= 0.0395. In E-G, *:p<0.05, as calculated by unpaired t test. **H)** Correlation between frequency of $CD8^+$ and proportion of tumor with immune niches in SOC and pSRS.

Supplemental Figure 4

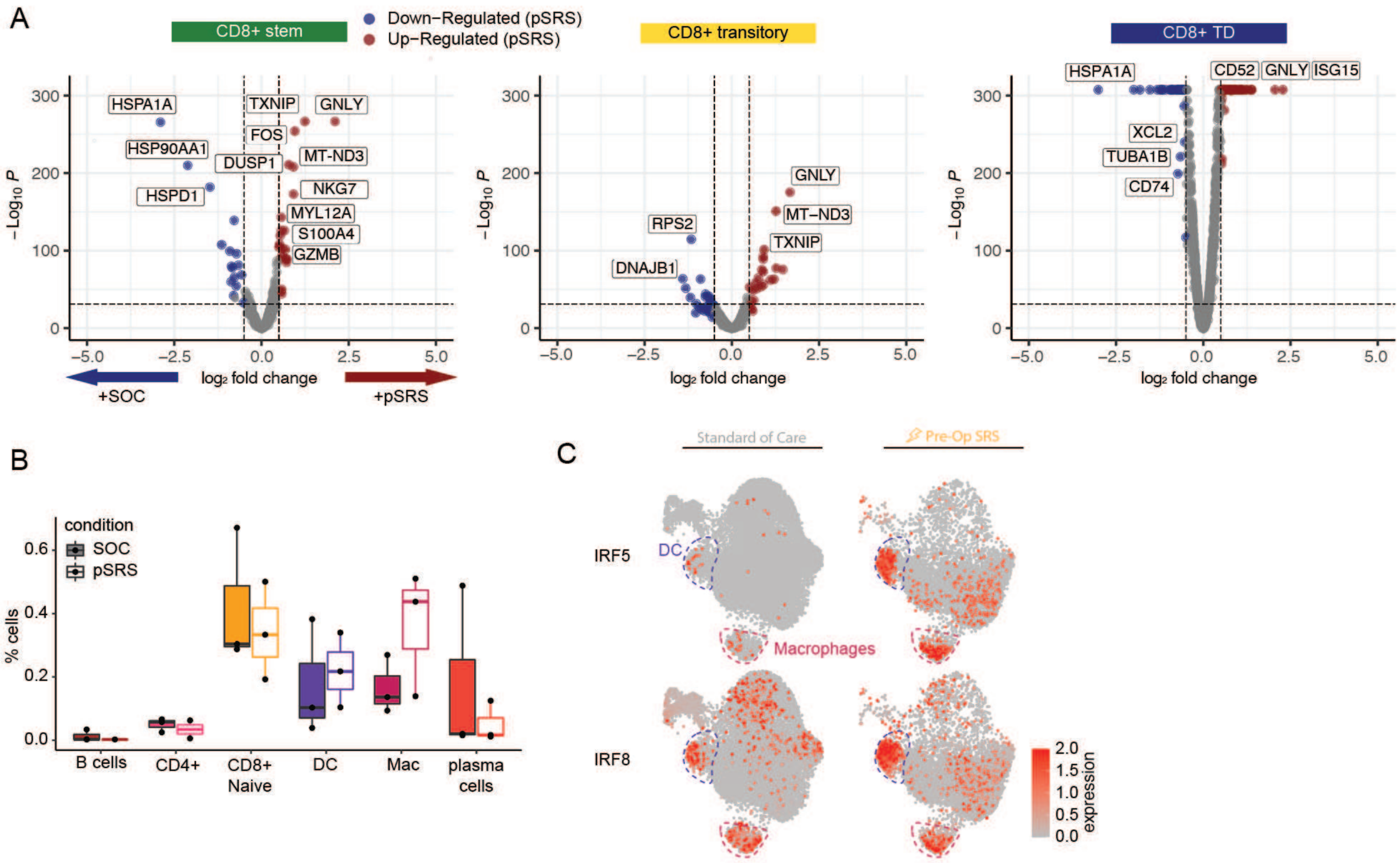

Supplemental Figure 4. A) Pairwise comparison of specific CD8$^+$ T cell clusters between SOC and pSRS. Volcano plots show average fold-change by all cells in the cluster by -log10 p-value. The number of differentially expressed genes (≥0.25 Log2 fold-change in each pairwise comparison) are indicated in each plot. **B)** Frequencies of various infiltrating immune cells compared between SOC and pSRS. **C)** Feature plots showing increased expression of IRF5 and IRF8 in DCs and macrophages in pSRS.

Supplemental Figure 5

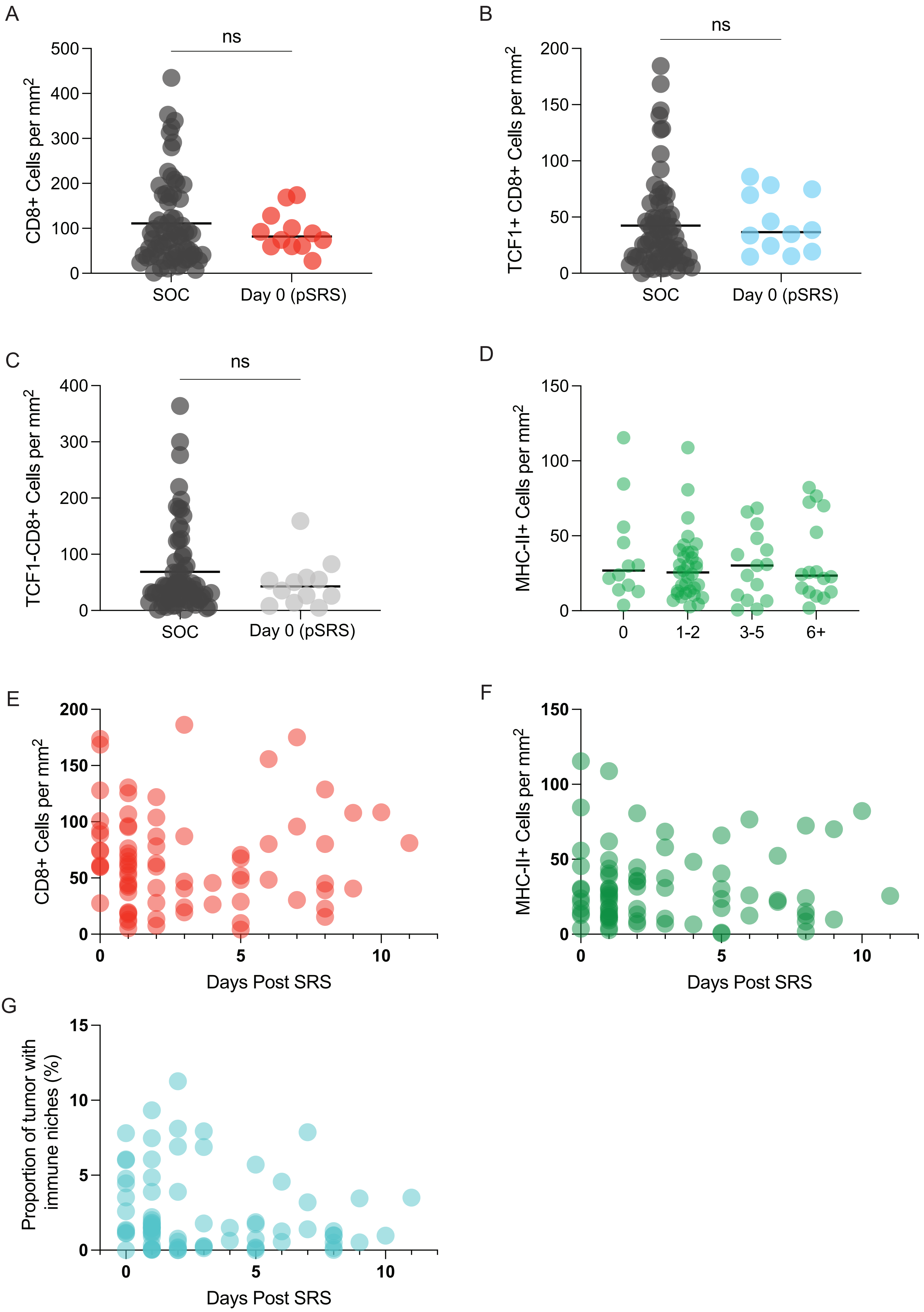

Supplemental Figure 5. **A)** SOC vs day 0 pSRS for CD8 per mm^2. **B)** SOC vs day 0 pSRS for $TCF1^+$ per mm^2. **C)** SOC vs day 0 pSRS for $TCF1^-$ per mm^2. A-C calculated by unpaired t test. **D)** $MHC\text{-}II^+$ cells per mm^2 show no change from day 0 to day 6+ after pSRS. **E**) Distribution of $CD8^+$ cells per mm^2 from 0-11 days after pSRS. **F)** Distribution of $MHC\text{-}II^+$ cells per mm^2 from 0-11 days after pSRS. **G)** Distribution of proportion of tumor with immune niches from 0-11 days post-SRS.
